# *Spiradiclisdetianensis* (Rubiaceae, Ophiorrhizeae), a new species from southwestern Guangxi, China

**DOI:** 10.3897/phytokeys.184.69886

**Published:** 2021-11-05

**Authors:** Zhao-Jie Wen, Yun-Fen Huang, Yan-Hua Hu, Khang Sinh Nguyen, Lei Wu

**Affiliations:** 1 College of Forestry, Central South University of Forestry and Technology, Changsha 410004, China Central South University of Forestry and Technology Changsha China; 2 Jiangxi Academy of Forestry, Nanchang 330032, China Jiangxi Academy of Forestry Nanchang China; 3 Guangxi Institute of Traditional Medical & Pharmaceutical Sciences,Nanning 530022, China Guangxi Institute of Traditional Medical & Pharmaceutical Sciences Nanning China; 4 Institute of Ecology and Biological Resources, Vietnam Academy of Science and Technology, 18, Hoang Quoc Viet Road, Cau Giay, Hanoi, 100000, Vietnam Institute of Ecology and Biological Resources, Vietnam Academy of Science and Technology Hanoi Vietnam

**Keywords:** China, Guangxi, limestone, Rubiaceae, taxonomy

## Abstract

A new species of Rubiaceae, *Spiradiclisdetianensis* is described from a limestone karst area of southwestern China. This new species is morphologically similar to *S.cordata* and *S.spathulata*. All of them have rosetted habit and long peduncles, but it differs from the former by the cuneate leaf bases (vs. basally cordate) and much longer corolla tubes (1.8–2.2 cm long vs. ca. 5 mm long), and from the latter mainly by its tubular-funnel shaped corolla (vs. slenderly salver shaped), 4.5–6.8 (vs. 1.5–2) mm in diam, inside throat and corolla densely puberulent (vs. glabrous except a ring of long hairs at the middle). It also resembles to *S.tubiflora*, but differs clearly by its subrosulate habit (vs. procumbent to creeping), longer leaf blades (7.0–10.5 cm vs. 0.5–2.5 cm) and longer corolla tubes (18–22 mm vs. 14–16 mm). At same time, color photos, illustrations, detailed descriptions and conservation status of the new species are provided.

## Introduction

*Spiradiclis* Blume is a morphological complex genus of Ophiorrhizeae (Rubiaceae). It is usually distinguished from its relatives by the subglobose or linear-oblong capsules with two or four twisted or straight valves when matured (Lo et al. 1983; Robbrecht 1988; Deb and Rout 1989; Lo 1999; Chen and Taylor 2011). In spite of the characteristic capsule form, the monophyly of the genus has been queried based on recent molecular evidence (Rydin et al. 2009; Razafimandimbison and Rydin 2019). Razafimandimbison and Rydin (2019) even reduced *Spiradiclis* and *Keenania* Hook.f. to the synonymy of *Ophiorrhiza* L. However, we find that the relationship between *Spiradiclis* and its relatives needs further research and thus we prefer to accept the traditional concept of *Spiradiclis* here, for the unique capsule form of the genus.

There are approximate 58 species of *Spiradiclis*, distributed in southeastern Asia and concentrated in southwestern China and northeastern India (Chen and Taylor 2011; Deng et al. 2014; Wang et al. 2015; Wen et al. 2015; Wu et al. 2015a, 2015b, 2016, 2019a, 2019b; Wang 2016a, 2016b; Pan et al. 2016, 2019; Liu et al. 2018; Zhang et al. 2018; Wen et al. 2019; Li et al. 2021). In China, 52 known species of *Spiradiclis* are recorded (Li et al. 2021).

Most representatives have a narrow habitat and prefer to grow at wet places on hill slopes or entrances of caves in limestone areas. During a field investigation to the neighboring regions between China and Vietnam in 2013, the second author came across a peculiar population of Rubiaceae. According to its calciphile habitat and rosetted habit, it is easy to associate with *Spiradiclis*. However, the flower shape of this population is very different from that of the known species of the genus. Its corollas are tubular-funnel shaped, with 1.8–2.2 cm long tubes, sharply shrunken near base, 4.5–6.8 mm in diam. at throat and 1.8–2.5 mm in diam. near the base, while the corollas of the genus are usually very short or slender. Hence, we revisited this population and collected its capsules. Their subglobose shape and dehiscence with 4 valves clearly indicate this population represents a species of the genus *Spiradiclis*. After careful comparisons with relevant literatures and examining specimens in herbaria, we found that our plant is most similar to *S.cordata* H.S. Lo & W.L. Sha and *S.spathulata* X.X. Chen & C.C. Huang, two species of subgenusSinospiradiclis, but it can be distinguished from the former by the cuneate leaf bases and much longer corolla tubes, and from the latter by its tubular-funnel shaped corolla and without a villous ring inside corolla. We conclude that this population represents an undescribed species and formally treat it here.

## Material and methods

Materials are deposited at the herbarium of Forest Plants in Central South University of Forestry and Technology (CSFI) and Guangxi Institute of Botany, Guangxi Zhuang Autonomous Region and Chinese Academy of Sciences (IBK) – herbarium acronyms follow Thiers (continuously updated). Morphological observations and measurements of the new species are based on living material in the field and dry specimens.

## Taxonomic treatment

### Spiradiclis detianensis

Taxon classificationPlantae GentianalesRubiaceae

L.Wu, Y.F.Huang & Z.J.Wen sp. nov.

A5D96615-0236-5760-9661-5F5DEFFFAE03

urn:lsid:ipni.org:names:77221569-1

Figs 1 , 2 A – I

#### Type.

China. Guangxi: Daxin county, Shuolong town, Detian village, 22°52'N, 106°43'E, elevation 650–750 m, 30 March 2018 (fl.), *Zhao-Jie Wen & Guang-Fu Mou 18033001* (holotype: CSFI [CSFI069613]; isotypes: CSFI, IBK).

#### Diagnosis.

The new species is similar to *Spiradicliscordata* and *S.tubiflora*, but it differs from the former mainly by the cuneate leaf base (vs. cordate) and much longer corolla tubes (1.8–2.2 cm long vs. ca. 5 mm long), and from the latter by its subrosulate habit (vs. procumbent to creeping), longer and wider leaf blades (7.0–10.5 × 2.0–3.5 cm vs. 0.5–2.5 × 0.4–1.5 cm), more secondary veins (7–10 pairs vs. 3–5 pairs) and longer corolla tubes (18–22 mm vs. 14–16 mm).

#### Description.

Perennial herb, up to 15 cm tall; stems densely pubescent, erect or ascending, lower part rooting at nodes. Leaves subrosulate; petiole 0.8–1.7 cm long; leaf blade drying papery, obovate-lanceolate or oblong, 7.0–10.5 × 2.0–3.5 cm, obtuse or acute at apex, cuneate at base, adaxially dark green, puberulent, abaxially light green, pubescent, densely pubescent along veins at lower surface; secondary veins in 7–10 pairs; stipules persistent, pubescent, narrowly triangular, 3.2–5.7 mm long, or 2–5-lobed, lobes linear-triangular, upper part filiform. Inflorescence cymose, 2–7-flowered; peduncle 9–14 cm long, pubescent; bracts linear-triangular, 4–6 mm long, subglabrous; pedicels 0.5–5 mm long, pubescent. Flowers distylous. Calyx puberulent; hypanthium portion obconic, ca. 2 mm long; lobes triangular, 1.2–2.9 mm long, acute at apex. Corolla white to pink, tubular-funnel shaped, puberulent outside; tube 1.8–2.2 cm long, sharply enlarged at the 1/3 lower part of the corolla tube, 4.5–6.8 mm in diam. at throat, 1.8–2.5 mm in diam. near base; lobes triangular-ovate, 4.5–5.6 × 3.5–4.3 mm long. Stamens 5; anthers linear. Stigma bilobed; ovary 2-celled. Long-styled flowers: corolla tube inside with densely pubescence near base and densely puberulent above anther and on to lobes; anthers inserted near base of corolla tube, 1.9–2.3 mm long; style 1.2–1.4 cm long, puberulent; stigma inserted at between middle and throat of corolla tube, 2-lobed, lobes elliptic, 1.8–2.2 mm long. Short-styled flowers: corolla tube inside densely puberulent; anthers inserted often at or a little above middle of corolla tube, 2.6–3.3 mm long; style 1.5–3.4 mm long, glabrous; stigma near base of corolla tube, lobes ovate-triangular, 1.3–1.6 mm long. Capsules subglobose, 2.5–3.2 mm in diam., valves 4 when matured. Seeds many, angular.

#### Phenology

. Flowering March to April, fruiting from May to July.

#### Etymology.

The specific epithet refers to the type locality, where a famous attraction, Detian Waterfall, is situated.

#### Chinese name.

德天螺序草 (de-tian-luo-xu-cao in Mandarin).

#### Distribution and habitat.

Until now, only two populations of the new species have been found. They are both known from limestone hills of southern Guangxi. Plants of the new species prefer to grow at humid places at elevation range of 500‒800 m, under evergreen broad-leaved forests with tree species of Fagaceae, Lauraceae, Tiliaceae, Theaceae, Myrsinaceae, Magnoliaceae and Sapindaceae.

**Figure 1.**
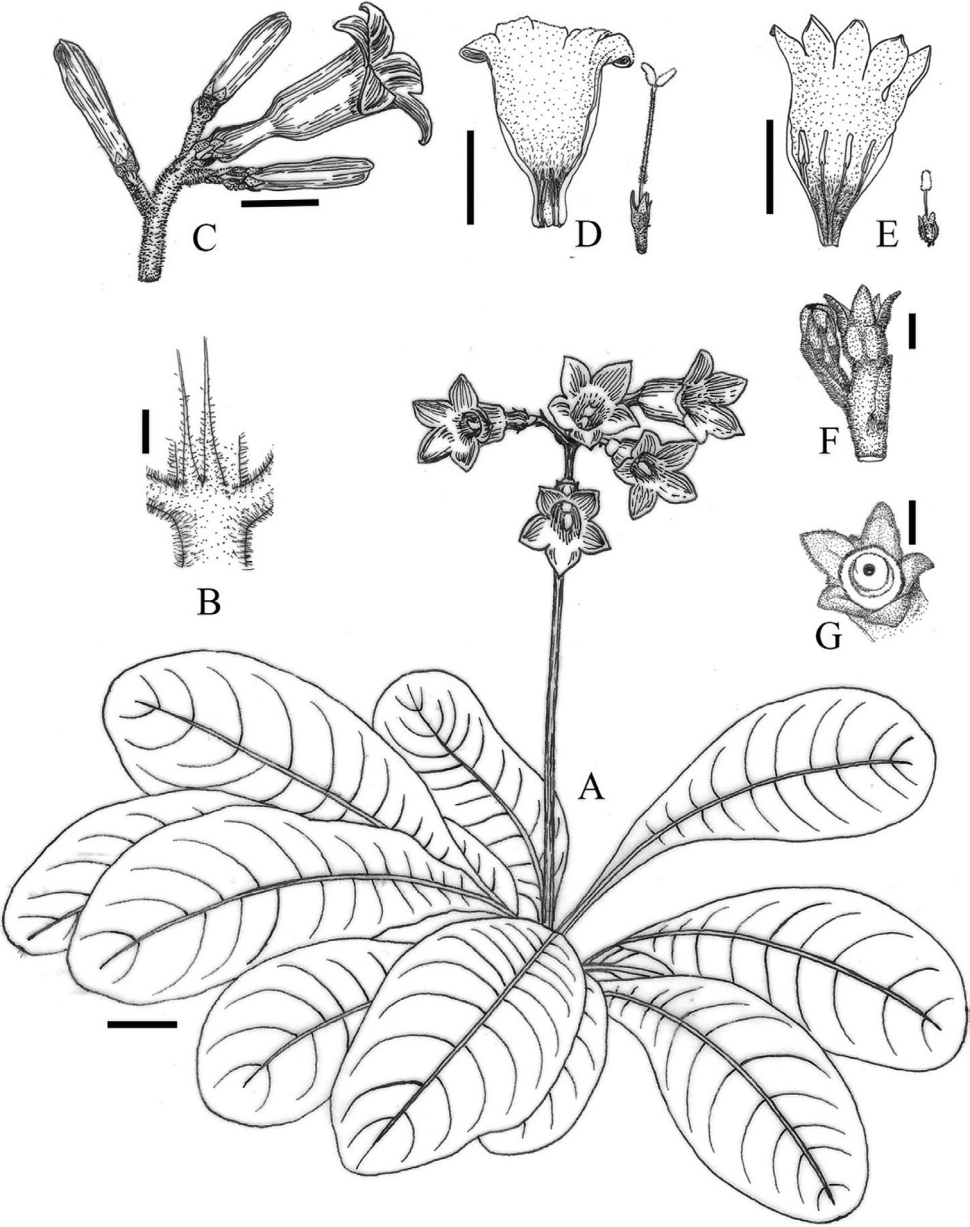
*Spiradiclisdetianensis***A** habit **B** stipule **C** inflorescence, side view **D** opened long-style flower **E** opened short-style flower **F** capsule, side view **G** capsule under matured, face view. Scale bar: 1 cm (**A, C, D, E**); 2 mm (**B, F, G**). Drawn from the holotype by X.Y. Zeng.

**Figure 2.**
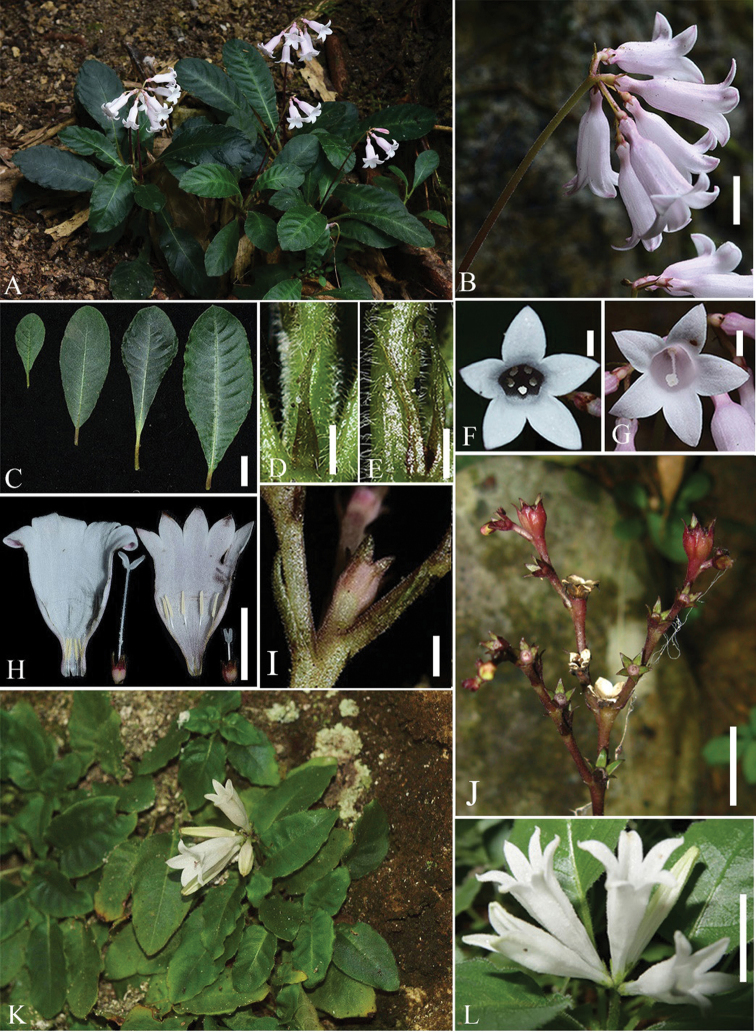
*Spiradiclisdetianensis***A** habit **B** inflorescence **C** leaves **D, E** stipule **F, G** short- and long-styled flower, front view **H** opened long- and short-styled flower, showing style and stamens **I** ovary, side view **J** infructescence, side view. *S.tubiflora***K** habit **L** inflorescence. Scale bars: 1 cm (**B, C, H, J, L**); 2 mm (**D, E, F, G, I**). Photos by Z.J. Wen and L. Wu.

#### Provisional conservation status

. During a series of field investigations at the China-Vietnam border over the past ten years (2009–2019), only three populations of *Spiradiclisdetianensis* have been observed. One site with nearly 59 matured individuals is distributed in Detian Waterfall (type locality), while the other two sites with more than 250 and 114 individuals are in the Longzhou county and Ningming county, respectively. The habitats of the three sites are in good condition and have been rarely influenced by humans. Considering the above, the species can be assigned a status of ‘Least concern’ [LC] following the guidelines of IUCN (2019).

#### Discussion.

Our unpublished molecular data indicates that *Spiradiclisdetianensis* shows the closest genetic relationship with *S.cordata*. Both species prefer to grow on limestone hills from southern Guangxi, China, and have similar habit, such as short stems, subrosulate leaves, long peduncles, heterostylous flowers, funnel-shaped corollas and subglobose capsules. However, the former can be easily distinguished from the latter mainly by its leaf blade basally cuneate (vs. basally cordate) and corolla tubes 1.8–2.2 cm long (vs. ca. 5 mm long) (more detailed comparisons are listed in Table 1).

**Table 1.** Morphological comparison of *Spiradiclisdetianensis*, *S.tubiflora* and *S.cordata* Lo et W. L. Sha.

	*Spiradiclisdetianensis*	*S.cordata*	*S.spathulata*	*S.tubiflora*
Habit	rosulate to subrosulate	rosulate to subrosulate	rosulate	procumbent to creeping
Leaf blade	obovate-lanceolate or oblong, 7.0–10.5 × 2.0–3.5 cm, base cuneate, apex obtuse or acute	elliptic-ovate to elliptic-oblong, 5–13 × 2–5.5 cm, base cordate, apex obtuse to rounded	spatulate or obovate-oblanceolate, 8–13 × 2–4.5 cm, base acute to cuneate, apex obtuse to rounded	ovate to elliptic, 0.5–2.5 × 0.4–1.5 cm, base rounded to obtuse, apex acute to rounded
Secondary vein	7–10 pairs	15–19 pairs	15–25 pairs	3–5 pairs
petiole	0.8–1.7 cm long	1–7 cm long	5–8 mm long	0.3–1.8 cm long
Stipule	triangular, entire or bifid	deeply 2-lobed, lobes linear	lanceolate-linear or linear	narrowly linear
Inflorescence	cymose, one per plant, 2–7-flowered	cymose to paniculate, 1–3 per plant, many flowered	cymose, 10– to many flowered	cymose, one per plant, 2–5-flowered
Peduncle	9–14 cm long	6–16 cm long	7–12 cm long	1.2–1.5 cm long
Bract	linear-triangular, 4–6 mm long	linear or subulate, 2 mm long	linear-lanceolate, 3–4 mm long	subulate, 1.8–3.0 mm long
Calyx lobe	triangular, 1.2–2.9 mm long	triangular, ca. 0.8 mm long	narrowly lanceolate, 1–1.3 mm long	triangular, 1.4–1.6 mm long
Corolla color	Purple	White	purple-reddish	white
Corolla tube	tubular-funnelform, sharply enlarged at the 1/3 lower part of the corolla tube, 18–22 mm long	tubular-funnelform, sharply enlarged at the middle or 1/3 upper part of the corolla tube, 5 mm long	slenderly salverform, tube 15–25 mm long	tubular-funnelform, slightly enlarged from the base to the throat, 14–16 mm long
Corolla inside (long-styled form)	without villous ring	with villous ring at middle	with villous ring at middle	Without villous ring

*Spiradiclisdetianensis* is very similar to *S.spathulata* in morphology, since both have subrosetted habit, obovate-oblanceolate leaf blades, long peduncles and subglobose capsules, but it differs mainly by its corolla tubular-funnel shaped (vs. slenderly salver shaped), 4.5–6.8 (vs. 1.5–2) mm in diam. at the middle and densely pubescence inside corolla near base and densely puberulent above anther and onto lobes (vs. glabrous except a ring of long hairs at the middle) (more detailed comparisons are listed in Table 1).

*Spiradiclisdetianensis* is a distinct species in the genus *Spiradiclis* due to the corolla tubes 18–22 mm long and having the shape of a reversed wine bottle, with an abruptly narrowed lower third, 4.5–6.8 mm in diam. at throat, 1.8–2.5 mm in diam. near base. Until now, only one other known species, *S.tubiflora* L.Wu, B.M.Wang & B.Pan (Wu et al. 2019b), has a similar corolla shape (see Fig. 2 K & L). However, *S.detianensis* differs from *S.tubiflora* principally by its rosulate habit (vs. procumbent to creeping), leaf blades longer than 7 cm and wider than 2 cm (vs. shorter than 2.5 cm and narrower than 1.5 cm), secondary veins 7–10 pairs (vs. 3–5 pairs) and longer corolla tubes (vs. 18–22 mm vs. 14–16 mm) (more detailed comparisons are listed in Table 1).

#### Additional specimens examined.

(paratypes). China. Guangxi: Longzhou county, Nonggang National Nature Reserve, 2 April 2019 (fl.), *Zheng-Quan Nong nzq0004* (CSFI); Niming county, Tingliang Town, Lixin village, 28 July 2011 (fr.), Yu-Song Huang 9422 (IBK).

## Supplementary Material

XML Treatment for Spiradiclis detianensis

## References

[B1] Chen T Taylor CM (2011) *Spiradiclis*. In: Wu ZY Raven PH (Eds) Flora of China, vol.19. Science Press, Beijing & Missouri Botanical Garden Press, St. Louis, 330–339.

[B2] Deb DB Rout RC (1989) Two new species of the genus *Spiradiclis* (Rubiaceae) from India. Candollea 44: 225–229.

[B3] Deng SJ Wen HZ Huang XX Wang RJ (2014) *Spiradicliscoriaceifolia* and *S.tonglingensis*, spp. nov. (Rubiaceae, Ophiorrhizeae) from Guangxi, China. Nordic Journal of Botany 5(5): 594–601. 10.1111/njb.00461

[B4] IUCN (2019) Guidelines for Using the IUCN Red List Categories and Criteria. Version 12. Prepared by the Standards and Petitions Subcommittee. http://www.iucnredlist.org/documents/RedListGuidelines.pdf [accessed: 18 March 2019]

[B5] Li JL Yuan Q Liu Y Song XF Pan B Qu CH Wu L (2021) Two new species of *Spiradiclis* (Rubiaceae) from limestone areas in southwestern China. Nordic Journal of Botany 39(2): e02979. 10.1111/njb.02979

[B6] Liu J Pan B Li SW Xu WB (2018) *Spiradiclisquanzhouensis* (Rubiaceae): A new species from limestone area in Guangxi, China. Nordic Journal of Botany 36(3): e01595. 10.1111/njb.01595

[B7] Lo HS (1999) *Spiradiclis* Blume. In: Lo HS (Ed.) Flora Reipublicae Popularis Sinicae.Vol. 71 (1). Science Press, Beijing, 86–110.

[B8] Lo HS Sha WL Chen XX (1983) A revision of the genus *Spiradiclis* Blume. Acta Botanica Austro Sinica 1: 27–36.

[B9] Pan B Ma HS Wang RJ (2016) *Spiradiclispengshuiensis* (Ophiorrhizeae, Rubioideae), a new species from Chongqing, China. PhytoKeys 63: 41–45. 10.3897/phytokeys.63.8016 PMC4956927 27489477

[B10] Pan B Tu RH Hareesh VS Wu L (2019) *Spiradicliscavicola* (Rubiaceae), a new species from Limestone Caves in Southwestern China. Annales Botanici Fennici 56(1–3): 1–4. 10.5735/085.056.0101

[B11] Razafimandimbison SG Rydin C (2019) Molecular-based assessments of tribal and generic limits and relationships in Rubiaceae (Gentianales): Polyphyly of Pomazoteae and paraphyly of Ophiorrhizeae and *Ophiorrhiza*. Taxon 68(1): 72–91. 10.1002/tax.12023

[B12] Robbrecht E (1988) Tropical woody Rubiaceae. Opera Botanica Belgica 1: 599–602.

[B13] Rydin C Kainulainen K Razafimandimbison SG Smedmark JEE Bremer B (2009) Deep divergences in the coffee family and the systematic position of *Acranthera*. Plant Systematics and Evolution 278(1–2): 101–123. 10.1007/s00606-008-0138-4

[B14] Thiers B (continuously updated) Index Herbariorum New York Botanical Garden’s Virtual Herbarium Database. http://sweetgum.nybg.org/ih/ [accessed: 10 June 2020]

[B15] Wang RJ (2016a) *Spiradiclisjingxiensis* sp. nov. (Rubiaceae) from Guangxi, China. Nordic Journal of Botany 34(5): 550–552. 10.1111/njb.01134

[B16] Wang RJ (2016b) *Spiradiclisyangchunensis* (Rubiaceae), a new species from Guangdong, China. Zhiwu Kexue Xuebao 34: 13–17.

[B17] Wang RJ Wen HZ Deng SJ Zhou LX (2015) *Spiradiclisdanxiashanensis* (Rubiaceae), a new species from South China. Phytotaxa 206: 30–36. 10.11646/phytotaxa.206.1.5

[B18] Wen HZ Wang RJ Deng SJ (2015) *Spiradiclislonganensis*, a new species of Rubiaceae from China. PhytoKeys 55: 113–117. 10.3897/phytokeys.55.4975 PMC4547028 26312046

[B19] Wen ZJ Yang JC Xu YF Wu L (2019) *Spiradiclisdensa* sp. nov. (Rubiaceae) from limestone areas in guangxi, china. Nordic Journal of Botany 37(6): e02190. 10.1111/njb.02190

[B20] Wu L Wang JL Liu QR (2015a) *Spiradiclispauciflora* (Rubiaceae), a new species from limestone areas in Guangxi, China. Annales Botanici Fennici 52(3–4): 257–261. 10.5735/085.052.0318

[B21] Wu L Wang JL Mo SS Liu QR (2015b) *Spiradiclisglandulosa* sp. nov. (Rubiaceae) from limestone areas in southern China. Nordic Journal of Botany 33(1): 79–82. 10.1111/njb.00577

[B22] Wu L Tong Y Pan B Liu QR (2016) *Spiradiclisglabra* sp. nov. (Rubiaceae) from limestone area in Guangdong, China. Nordic Journal of Botany 34(6): 718–721. 10.1111/njb.01156

[B23] Wu L Li X Liu WJ Liu QR (2019a) *Spiradicliskarstana* (Rubiaceae), a new species from Yunnan, China. PhytoKeys 117: 1–8. 10.3897/phytokeys.117.28281 PMC6370749 30766422

[B24] Wu L Wang BM Pan B Yu XL (2019b) *Spiradiclistubiflora* (Rubiaceae), a new cave-dwelling species from southern China. PhytoKeys 130: 217–224. 10.3897/phytokeys.130.34625 31534408 PMC6728394

[B25] Zhang F Liu Y Wen ZJ Wu L (2018) *Spiradiclislui*, a new species of Rubiaceae from Guangxi, China. Nordic Journal of Botany 2018(6): e01786. 10.1111/njb.01786

